# Use and Perception of Contraception among Genders in Santo Domingo, Dominican Republic

**DOI:** 10.5334/aogh.2430

**Published:** 2019-06-24

**Authors:** Rebecca Khamishon, Jiabi Chen, Naomie Ranatunge, Qianni Wu, Nicole Downey, Eleanor Love, Jeidi Garcia Rodriguez, Mark Ryan

**Affiliations:** 1 VCU School of Medicine 2021, US; 2 VCU School of Pharmacy 2020, US; 3 VCU School of Nursing 2019, US; 4 VCU School of Pharmacy 2021, US; 5 VCU Department of Family Medicine and Population Health, US

## Abstract

**Background:**

The prevalence of contraception in the Dominican Republic is among the highest of Latin American countries. Prior research has assessed the general perception of contraception in Latin America, examined determinants of contraceptive use among Dominican women, and explored their perceived reproductive control. Little research has explored the specific role each sexual partner, male and female, has in determining the use of contraception in Latin American countries.

**Objective:**

This study aims to address the gap in research regarding the specific role each sexual partner has in determining the contraception use in Latin American countries by evaluating male and female perception and use of contraception, and their perceived reproductive control.

**Methods:**

A one-time survey was administered to adult patients of two short-term medical missions located in Santo Domingo, Dominican Republic. The difference in overall responses to dichotomous questions and ordinal questions were tested using binomial Z-test and nonparametric Chi-Square Goodness-of-Fit test. Bivariate analyses were conducted using cross tabulation with Chi-Square test.

**Findings:**

The majority of participants of both genders are in favor of contraception use, believe they have the power to avoid an unplanned pregnancy, and view their partners’ wishes regarding the use of contraception as important. However, significantly more females than males are in favor of contraception use (p-value = 0.01). Specific subgroups of men and women answered the survey in ways that suggest traditional values may be at odds with individuals’ willingness to use contraception.

**Conclusions:**

There is an overall acceptance of contraception use and perception of reproductive control among both genders in our population, with a greater proportion of females in favor of contraception use than males (p-value = 0.01). Changing cultural norms may be coming into conflict with established beliefs and practices in the Dominican Republic, such as its *machismo* culture.

## Introduction

The prevalence of contraception in the Dominican Republic, at 71.8%, is among the highest of Latin American countries and nearly comparable to the prevalence in the United states (75.1%) [1,2]. Though contraception is widely available in the Dominican Republic, the rate of teenage pregnancy and maternal mortality are notably high, and the teenage pregnancy rate is rising [3]. In 2015, the country’s government launched an initiative to require sexual education in all public schools [4], which has equipped young adults with more knowledge about sexual health and contraception methods [5].

Prior research has assessed the general perception of contraception in Latin American countries, examined determinants of contraceptive use among Dominican women, and explored Dominican women’s perceived reproductive control [1,6]. A prior study conducted in Santiago found that women are expected to take responsibility for contraception due to their male partner’s unwillingness to do so [6]. The patriarchal culture of the Dominican Republic creates a society that traditionally favors male dominance [7]. This *machismo* culture, with its emphasis on traditional gender roles and female subordination, may be attributed to and sustained by strong Roman Catholic influence that emphasizes families over individuals as the fundamental unit of society. In marriage, sexuality, and other aspects of Dominican culture, Catholic values perpetuate the paternalistic attitude [8].

Little research has probed the specific role each sexual partner (male/female) has in determining the overall usage of birth control in Latin American countries. This research aims to address this gap by: (1) exploring how the perception of contraception differs between genders in the communities of Paraiso and Los Mina in Santo Domingo, Dominican Republic; and (2) investigating how men and women feel their beliefs versus their partners’ beliefs impact decisions about contraceptive use as well as their perceived reproductive control. Ultimately, this research aims to inform future interventional programs that provide contraception education and resources, while advancing the understanding of barriers to contraception use among adults in the Dominican Republic.

## Methods

### Participants

A cross-sectional survey was conducted among adult patients of two short-term medical missions (STMM) located in Santo Domingo, Dominican Republic in June 2018. The clinics were located in the Paraiso and Los Mina sectors of Santo Domingo. These STMM clinics are part of biannual medical service trips to the Dominican Republic sponsored by the Humanitarian Outreach Medical Brigade Relief Effort (HOMBRE) and the Dominican Aid Society of Virginia (DASV), both US-based 501c3 non-profit organizations, in collaboration with non-profit Dominican-based medical service organizations. All patients between the ages of 18 and 89 were invited to participate in the survey (n = 378). Children under 18 and individuals over 89 were excluded due to privacy considerations. This study was approved by the Virginia Commonwealth University Institutional Review Board (IRB) in Richmond, Virginia (HM20013031).

### Survey Instrument

Participants were informed of survey administration through public announcement made at the beginning of clinic. Subjects were approached individually to be recruited while waiting for medical care. If a subject expressed interest in participating, they were taken to a private room for survey administration. A standardized verbal consent was read to the participant. Age was obtained to ensure the participant was between the age of 18 and 89, and participants were read the survey one question at a time. No identifiers were collected for this study. The surveys were administered by functionally bilingual members of the research team or in collaboration with Dominican medical students or professionals serving as volunteer interpreters.

**Figure 1:**
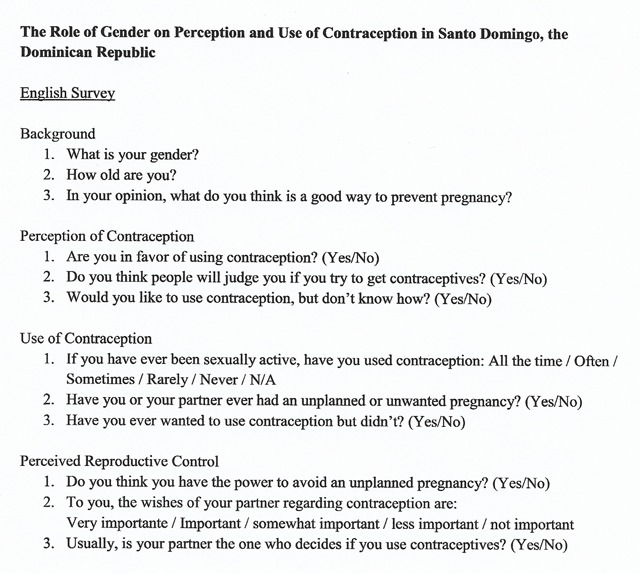
Quantitative cross-sectional survey administered in the Dominican Republic.

A quantitative cross-sectional survey consisting of 12 questions was created based on key survey questions from DeGette et al., 2014 (adapted with permission) to evaluate subjects’ perception and use of contraception, and their perceived reproductive control. The survey documented key demographics, perceptions and use of contraception, and perceived reproductive control. A copy of the survey is included in Figure 1. One survey question (i.e., “Would you like to use contraception but don’t know how?”) was excluded from analyses due to risk of misinterpretation. When translated, the question was interpreted as two separate questions, and it was unclear which part of the question the participants were answering.

### Statistical Analysis

The difference in overall responses to dichotomous questions and ordinal questions were tested using binomial Z-test and nonparametric Chi-Square Goodness-of-Fit test using SPSS (©IBM Inc. Version 25, 2017). Bivariate analyses were conducted using cross tabulation with Chi-Square test demonstrating relationships between all dichotomous/categorical variables first for the whole sample of participants (male + female combined), and for male and female subsamples individually. Results of all statistical tests with a p-value < 0.05 were considered statistically significant and reported. The binomial 95% confidence intervals (CIs) of proportion of answering “yes” to dichotomous survey questions were calculated for both male and female populations based on the estimation from our samples using Sample Size Calculators (UCSF Clinical & Translational Science Institute, 2018). Results from individuals who did not finish the survey were also included in the analyses. As each question is independent from one another, it does not affect the qualification of other responses. When calculating percent for each question, only valid percent was reported and missing responses were not counted. Age of participants was stratified into four age groups (15–24, 25–44, 45–64, 65+) and a subgroup analysis was conducted. Participants’ answer to Q3 (“In your opinion, what do you think is a good way to prevent pregnancy?” were categorized into: 1. Abstinence; 2. Contraceptives [e.g., condom, pills]; 3. Contraceptives and family planning; 4. Others; 5. “I don’t know”; 6. Sterilization & contraceptives and/or family planning; 7. Education).

## Results

One hundred forty-three individuals met the criteria and participated in the study. All responses from individuals who met the age qualification (18–89) are included in the analyses. Common reasons for non-participation were: ineligible due to age, patient concerns about time commitment, and a perception of inconvenience of completing the survey. The survey results were analyzed within the categories of male gender, female gender, and as a whole. Out of the 143 total participants, the age range was from 18–86 years old, with 43 males (30.1%) and 100 females (69.9%) included in the study. Overall, the median age was 52 years old and the mean age was 49. Within the male category, the mean age was 53 and within the female category, the mean age was 47 (Table 1).

**Table 1:** Demographic information of survey participants.

Characteristic	Overall	Male	Female

**Gender N (%)**			
**Male**	43 (30.1)	–	–
**Female**	100 (69.9)	–	–
**Age**			
**Mean (SD)**	49.27 (16.09)	53.49 (16.13)	47.45 (15.8)
**Median**	52.0	55	49.5
**Range**	18–86	18–86	18–78
**Age group N (%)**			
**18–24**	12 (8.4)	2 (4.7)	10 (10)
**25–44**	38 (26.6)	9 (20.9)	29 (29)
**45–64**	66 (46.2)	22 (51.2)	44 (44)
**65+**	27 (18.9)	10 (23.3)	17 (17)

**Figure 2:**
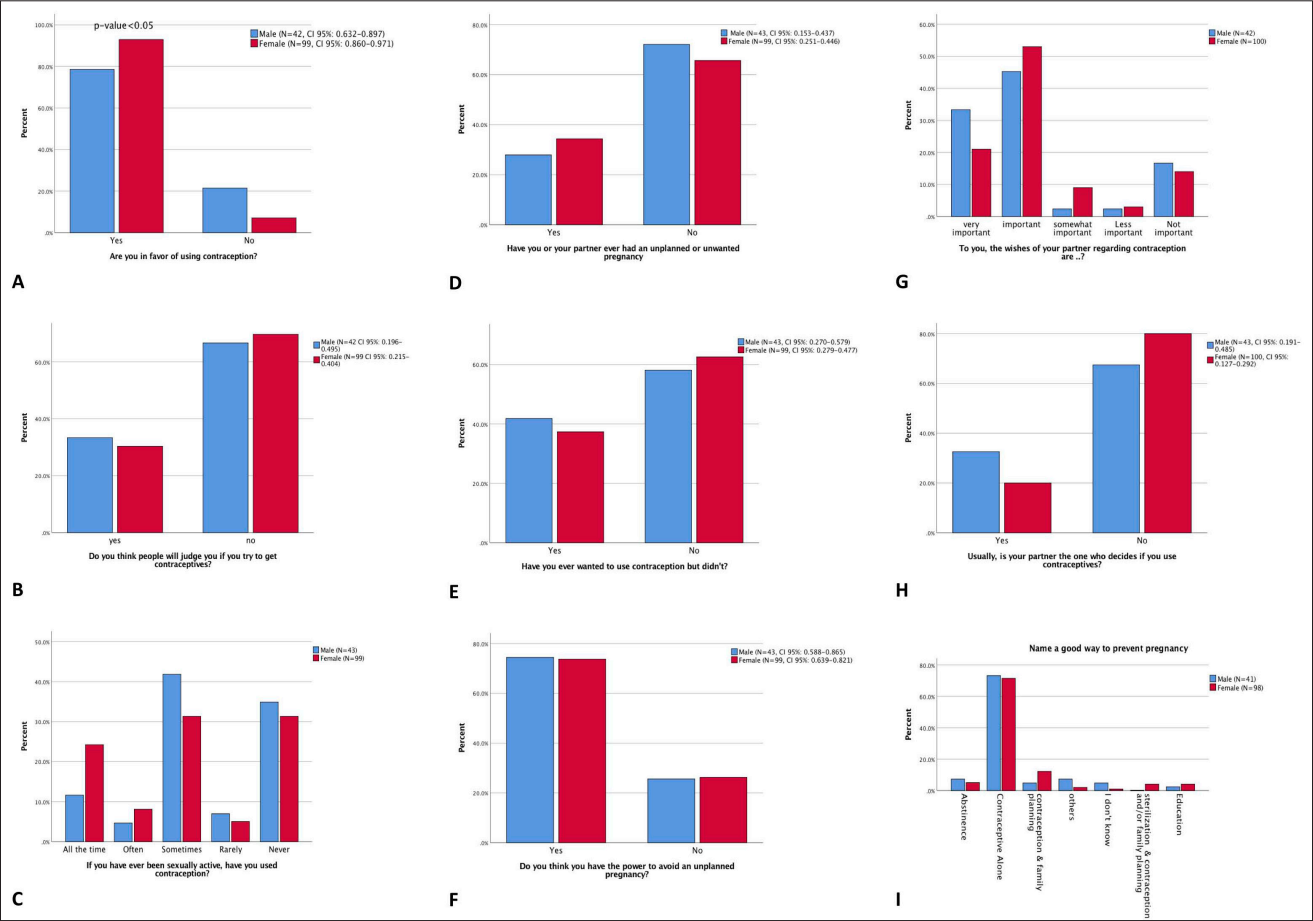
**Survey responses breakdown by genders. (A)–(I)** are bar charts survey responses showing in percent and grouped in genders. Cross tabulation with chi-Square analyses were performed on genders and each question to show association. The 95% confidence intervals of proportions who responded “yes” to dichotomous questions were reported for both genders.

Overall, female and male sample sizes and frequencies of each question are shown in Figure 2. Missing responses were also reported. Chi-Square analysis was conducted between male and female responses for each question. The survey responses were also presented in bar graphs on which 95% CI was included (Figure 2). The only question that is showed gender difference is “Are you in favor of using contraception?” (p-value = 0.01) (Figure 2A). Overall, both male and female participants are in favor of using contraception (88.7%, CI 95%: 0.822–0.934). A statistically significant difference was evident between genders, with females (92.9%, CI 95%: 0.860–0.971) more in favor than males (78.6%, CI 95%: 0.632–0.897) (Figure 2A). The remainder of the survey results did not present gender differences (Figure 2B–I).

Among all participants, statistically significant correlations were found between responses to the question “Are you in favor of contraception?” and responses to four other questions which address types of contraception, personal use of contraception, control of contraception use, and the importance of partners’ wishes regarding contraception use (Table 2).

**Table 2:** Correlation between responses to whether in favor of contraception use and responses to other questions from all participants.

	In favor of using contraception (N = 125)	Not in favor of using (N = 16)	p-value

**Name a good way to prevent pregnancy**			**<0.001**
Abstinence	6 (4.9%)	2 (13.3%)	
Contraceptive alone	95 (77.9%)	5 (33.3%)	
Contraception & family planning	12 (9.8%)	2 (13.3%)	
Others	3 (2.5%)	2 (13.3%)	
I don’t know	0 (0.0%)	2 (13.3%)	
Education	2 (1.6%)	2 (13.3%)	
Sterilization & contraception and/or family planning	4 (3.3%)	0 (0.0%)	
**If you have ever been sexually active, have you used contraception?**			**0.001**
Use All the Time	29 (23.4%)	0 (0.0%)	
Often	8 (6.5%)	2 (12.5%)	
Sometimes	48 (38.7%)	1 (6.3%)	
Rarely	7 (5.6%)	1 (6.3%)	
Never	32 (25.8%)	12 (75.0%)	
**Do you think you have the power to avoid an unplanned pregnancy?**			**0.003**
Yes	98 (78.4%)	7 (43.8%)	
No	27 (21.6%)	9 (56.3%)	
**To you, the wishes of your partner regarding contraception are…?**			**0.002**
Very Important	32 (25.6%)	3 (20.0%)	
Important	68 (54.4%)	3 (20.0%)	
Somewhat Important	9 (7.2%)	1 (6.7%)	
Less Important	3 (2.4%)	1 (6.7%)	
Not important	13 (10.4%)	7 (46.7%)	

Cross tabulation with Chi-Square analyses were performed between responses “Are you in favor of using contraception?” and responses to other questions given by all participants (N = 143).

In a subgroup analysis among female respondents categorized by age, four responses were significantly associated with age, three of which were also found to have significant associations with age groups of all participants regardless of gender (Table 3 & Appendix Table 1). Responses to any survey questions were significantly differed among age groups in men.

**Table 3:** Age study on survey responses from female participants.

	Age group				p-value

	18–24 (N = 10)	25–44 (N = 29)	45–64 (N = 44)	65+ (N = 17)	

**Do you think people will judge you if you try to get contraceptives?**					**0.015**
Yes	1 (10.0%)	10 (34.5%)	9 (20.9%)	10 (58.8%)	
No	9 (90.0%)	19 (65.5%)	34 (79.1%)	7 (41.2%)	
Missing	0	0	1	0	
**If you have ever been sexually active, have you used contraception?**					**0.023**
All the time	4 (40.0%)	7 (25.0%)	8 (18.2%)	5 (29.4%)	
Often	0 (0.0%)	5 (17.9%)	2 (4.5%)	1 (5.9%)	
Sometimes	4 (40.0%)	12 (42.9%)	14 (31.8%)	1 (5.9%)	
Rarely	1 (10.0%)	1 (3.6%)	3 (6.8%)	0 (0.0%)	
Never	1 (10.0%)	3 (10.7%)	17 (38.6%)	10 (58.8%)	
Missing	0	1	0	0	
**Have you ever wanted to use contraception, but did not?**					**<0.001**
Yes	8 (80.0%)	14 (50.0%)	15 (34.1%)	0 (0.0%)	
No	2 (20.0%)	14 (50.0%)	29 (65.9%)	17 (100.0%)	
Missing	0	1	0	0	
**To you, the wishes of your partner regarding contraception are…?**					**0.006**
Very important	1 (10.0%)	4 (13.8%)	11 (25.0%)	5 (29.4%)	
Important	4 (40.0%)	21 (72.4%)	23 (52.3%)	5 (29.4%)	
Somewhat important	4 (40.0%)	2 (22.2%)	3 (33.3%)	0 (0.0%)	
Less Important	0 (0.0%)	0 (0.0%)	2 (4.5%)	1 (5.9%)	
Not important	1 (10.0%)	2 (6.9%)	5 (11.4%)	6 (35.3%)	
**What do you think is a good way to prevent pregnancy?**					**0.056**
Abstinence	0 (0.0%)	0 (0.0%)	3 (7.0%)	2 (11.8%)	
Contraceptive alone	6 (60.0%)	25 (89.3%)	31 (72.1%)	8 (47.1%)	
Contraception & family planning	3 (30.0%)	3 (10.7%)	4 (9.3%)	2 (11.8%)	
Others	1 (10.0%)	0 (0.0%)	1 (2.3%)	0 (0.0%)	
I don’t know	0 (0.0%)	0 (0.0%)	1 (2.3%)	0 (0.0%)	
Sterilization and contraception and/or family planning	0 (0.0%)	0 (0.0%)	1 (2.3%)	3 (17.6%)	
Education	0 (0.0%)	0 (0.0%)	2 (4.7%)	2 (11.8%)	

Cross tabulation with Chi-Square analyses were performed between age groups and survey responses among female participants (N = 100).

A correlation study was performed on responses from males. Questions addressing contraceptive use and the locus of control in making decisions regarding contraception were found to be correlated with male’s responses to whether they were in favor of using contraception (Table 4).

**Table 4:** Correlation between responses to whether in favor of contraception and responses to other questions among male participants.

	Favor Contraception (N = 33)	Not Favor Contraception (N = 9)	p-value

**Usually, is your partner the one who decides if you use contraceptives?**			**0.017**
Yes	14 (42.4%)	0 (0.0%)	
No	19 (57.6%)	9 (100.0%)	
**To you, the wishes of your partner regarding contraception are…?**			**0.004**
Very important	12 (36.4%)	2 (25.0%)	
Important	18 (54.5%)	1 (12.5%)	
Somewhat important	0 (0.0%)	1 (12.5%)	
Less important	1 (3.0%)	0 (0.0%)	
Not important	2 (6.1%)	4 (50.0%)	
**If you have ever been sexually active, have you used contraception?**			**0.001**
All the time	5 (15.2%)	0 (0.0%)	
Often	2 (6.1%)	0 (0.0%)	
Sometimes	18 (54.5%)	0 (0.0%)	
Rarely	2 (6.1%)	3 (7.1%)	
Never	6 (18.2%)	14 (33.3%)	

Cross tabulation with Chi-Square analyses were performed between responses to “Are you in favor of using contraception?” and responses to all other questions given by male participants (N = 43).

## Discussion

The results of this study identify major topics of consideration regarding the perception of contraception among men and women. Overall, there were no gender differences identified in responses to most of the survey questions with the exception that more women than men indicated favorability toward contraception use. The majority of respondents regardless of gender believe they have the power to avoid an unplanned pregnancy. In addition, the majority of participants also view their partners’ wishes regarding the use of contraception as being either very important or important, but state that they themselves decide whether or not to use contraception. The majority of participants do not think they will be judged by society if they use contraception, and an even greater majority are in favor of contraception use. However, there may be a minority of the population that would like to use contraception but believe judgement would be an issue.

Overall, this data suggests a general acceptance of contraception by both genders among the population of one community in the Dominican Republic. It is possible that individuals from the community of the present study, especially women, are gaining confidence and becoming more aware of their autonomy in using contraception. Growing participation in wage employment has emboldened women in household decision making [9], which may translate to sexual relationships as well. It is possible that the relationship between heterosexual partners is becoming more equal and less dominated by men, which may allow for women to more effectively act on their preferences.

Participants who indicated a preference for using contraception were more likely to have previously used contraception at least “sometimes”. Also, they were more likely to be able to name at least one method of contraception when asked to name a way to prevent pregnancy at the beginning of the survey and were more likely to believe they have the power to avoid unplanned pregnancy. These results suggest that people who are in favor of contraception use are more likely to have used it before and have a good understanding of its value in preventing unplanned pregnancy. We can further extrapolate that because reproductive health policies have been liberalized in countries that are predominantly Catholic, particularly in Latin America, contraception has become more available and commonplace [10].

Although contraception has been gaining acceptance in a society of the Dominican Republic, our results suggest that there still may be gaps in its access and effective use. Nearly one-third of respondents stated that they have never used contraception, and a similar number of respondents marked “yes” when asked if they or their partner have ever had an “unplanned or unwanted pregnancy”. There is no statistical significance in the correlation between these two responses, which may indicate that people who state that they have used contraception before could still have an unplanned pregnancy. This may result from ineffective usage of contraception or lack of access to contraception. In addition, the high acceptance and favorability of using contraception reflected in our results does not explain the high teenage pregnancy rate in Dominican Republic. According to the 2017 National Human Development Report for the Dominican Republic, 22% of women between ages 12 and 19 have been pregnant, a rate that is 34% higher than average for Latin America and Caribbean nations. The same report further asserts that the high teenage pregnancy rate is concentrated in poorer communities, which may be explained by a lack of access to contraception or sexual health education [11]. A potential barrier, regardless of the generally positive attitude toward contraception, may be a lack of education or consistent, reliable access. Because our study only addressed the perceptions of women ages 18 and older, it should be considered that the views of women between the ages of 12 and 19 could have significantly impacted the results of the study. This limitation provides an opportunity for future research to explore possible differences in views regarding acceptance of contraception usage in women above versus below the age of 18, and if this could help explain the high teenage pregnancy rate in the Dominican Republic. Regardless, this issue opens the door for further research, and these gaps in understanding offer opportunities for governments, health systems, and future short-term medical missions to make contraception and sex education more available.

Another consideration from the results is the potential demographic shift in the attitude of the people in the Dominican Republic regarding sex and the influence of traditional values. Our results reveal that women in the 65+ years age range were more likely to indicate that people will judge them if they try to get contraceptives compared to other age groups of females; additionally, women in this age range are more likely to indicate they do not think they have the power to avoid unplanned pregnancy (Table 3). The sentiments of older women as reflected in the results may indicate that judgement from society was a barrier to using contraception, rather than the wishes of their partners. Previous studies indicate that cultural factors, such as the power imbalance of the *machismo* culture, could contribute to access-independent low reproductive control [6]. Our results suggest that the lack of contraception use among older women was more due to judgement from society versus male partner influence. When asked about ways to prevent pregnancy, women from older age groups were more likely to respond with options such as “family planning” or “sterilization” while younger women were more likely to indicate specific forms of contraception, such as “the pill” or “condoms”. These points suggest that the newer generation of women in the Dominican Republic may be becoming more independent and powerful in decisions regarding their sexuality, as previously indicated with the newfound power in the workforce [12], in addition to being more knowledgeable about the wide range and specific forms of contraception available to them.

While women’s responses indicated a shift in the distribution of power among the population, our results also revealed a potential preservation of the *machismo* culture in the Dominican Republic. This patriarchal culture traditionally favors male dominance and has an emphasis on traditional gender roles and female subordination which may be attributed to a Roman Catholic influence [7,8]. Male participants who indicated that they do not favor the use of contraception were also more likely to indicate that their partner’s wishes were not important and less likely to say their partner is the one who decides if they use contraception. This could be an indicator of the lingering machismo culture in the Dominican Republic, where “male supremacy see women in a socially disadvantaged position, where it is understood that their proper space is in the home” [13]. Although men made up a minority of the sample size (n = 43 of 143), they may significantly impact family members and sexual partners. The significance of just a small percentage of male participants who are not in favor of contraception indicating their partner’s wishes as not important (versus a *machismo* culture where this view might be a majority among men) may be due to male dominance in Latin America being disputed by social actions in recent years. Men and masculinities have been impacted in dramatic ways by feminist projects in which men have been challenged by women’s independence and initiative [13]. These results leave room for future studies to explore views among men in the Dominican Republic who do not favor the use of contraception, and to determine if there is a similar trend among other Latin American cultures. Further investigation could explore and analyze whether different demographic factors, such as age and socioeconomic status, influence men’s perceptions of contraception.

The conducted research did have some limitations. Participants of the current study are predominantly females which might skew the results due to its gender imbalance, the study was restricted to Santo Domingo and thus its results might have limited geographic representation, and there was one question that posed risk of misinterpretation which was ultimately excluded from the results. Some surveys were not completed; however, the data was still used given that survey questions were independent of each other. Regardless, no single question was omitted more than any other, indicating there was not a singular unfavorable question. Another limitation was that other than comparing men’s and women’s responses, subgroup analyses were not pre-specified, and some of the subgroups had small numbers of included answers. This may lead to a risk of overstating these results.

Ultimately, this research conducted in Santo Domingo may represent a microcosm of the Dominican Republic and potentially Latin America as a whole. The results of our research reveal a wide acceptance in contraception, gaps in its use, and a potential shift in the prevalence of traditional, conservative values. Our results highlight similar findings as those from a prior study conducted among women in Santiago [6]. In Santiago, the results of the survey indicated that even with “the power imbalance of the *machismo* culture, [women] will take responsibility for contraception based on men’s reluctance to use contraceptives” [6]. This prior research in Santiago parallels our results in Santo Domingo, indicating that in the Dominican Republic, overall acceptance of contraception is not an issue, and women may be the ones who hold the responsibility in its usage. In Santiago, societal factors contributed to women being less likely to use contraception [6], which aligns with our results that some women fear judgement from society if they try to access contraceptives. Our research opens up new avenues to further investigate perceptions of reproductive autonomy for both men and women as well as shifts in cultural and societal values.

## Conclusion

This research aimed to explore how the perception of contraception differs between genders in the communities of Paraiso and Los Mina in Santo Domingo, Dominican Republic. Based on this study, a greater proportion of women indicated that they favor the use of contraception, but both men and women overall favor the use of contraception. No significant gender differences were noted for other survey questions. These results also suggest that individuals in Paraiso and Los Mina have confidence in contraception and are aware of their autonomy in its use. The majority of both men and women respondents believe they have the power to avoid unplanned pregnancy, view their partners’ wishes of using contraception as important, and state that they themselves decide whether or not to use contraception. The results of our current study parallel those of previous research conducted in Santiago, Dominican Republic, revealing that the overall acceptance of contraception is not an issue [6]. Nevertheless, our results reflect that there still may be gaps in the access and the effective use of contraception. In addition, different opinions based on age and gender suggest that some changing cultural norms may be coming into conflict with established beliefs and practices.

Overall, the results of this research have the potential to inform future interventional programs that provide contraception education and resources by enhancing the current understanding regarding barriers to contraceptive use among adults in the Dominican Republic.
